# Study on lean production management of new energy vehicle body painting based on the dual perspectives of digital transformation and VSM

**DOI:** 10.1371/journal.pone.0318253

**Published:** 2025-02-14

**Authors:** Qin Yang, Xizhe Wang, Hu Wu

**Affiliations:** 1 College of Business, Gachon University, Seongnam, Republic of Korea; 2 School of Mechanical Engineering, Shandong University of Technology, Zibo, Shandong, China; King Khalid University, SAUDI ARABIA

## Abstract

The automobile body painting production system exists in the backward management concept of focusing on production and ignoring environmental protection, focusing on traditional technology and unwilling to innovate, etc., high production cost and environmental protection standards, which have had a negative impact on the industry’s long-term growth. Based on the value stream mapping (VSM) analysis technology, the current situation of the value stream of the body spraying process of TJ Automobile Manufacturing Company is analyzed, the problem of waste in the production process of automobile body painting system affected by Eliminate Combine Rearrange Simplify (ECRS)a four-step problem-solving technique was studied and analyzed, a new method for the application of value stream graph analysis technology in the digital transformation environment is proposed, and a suitable production improvement scheme for the body painting system is formulated by using the new method, which increases the value-added operation time of the painting production process by 9.5% and reduces the non-value-added operation time by 12.5%. The number of operators was reduced by 18.8%, the value-added ratio was increased accordingly, the scrap rate was reduced by 8%, and the exhaust gas purification rate was increased by 66%, which brought good economic and social benefits to the enterprise and provided a successful case for the application of lean production VSM in the digital transformation environment. The paper extends knowledge in the area of digital transformation and lean production by helping us to establish and explain the relationship between lean production management methods and the promotion of new energy vehicle body coating performance from a dual perspective of digital transformation and VSM. None of the previous studies have considered the combined application of the studied digital transformation and VSM lean management approaches.

## 1. Introduction

Around the world, energy shortages and environmental degradation have become serious challenges, the development of electric vehicles is a crucial measure to alleviate the contradiction between world fuel supply and demand, reduce emissions of exhaust pollutants, improve the quality of the atmospheric environment, and promote continuous technological progress and optimization and upgrading of the automotive industry. In 2010, China designated the electric vehicle industry as a strategic emerging industry. In 2015, it introduced the new development concept of “innovation, coordination, green, openness, and sharing,” and in November 2020, it released the “New Energy Vehicle Industry Development Plan (2021–2035),”which clearly set the 2025 development goal of achieving about 20% of new car sales volume for new energy vehicles and the long-term goal of “by 2035, the core technology of China’s new energy vehicles should reach an internationally advanced level, and the quality and brand should have strong international competitiveness” [1,2]. At present, many electric vehicles paint body production lines are derived from the transformation of traditional light vehicle paint body production lines, with problems such as heavy pollution, high costs, and low levels of automation, and the industry’s competitiveness is intensifying [3]. At the same time, the quality of electric vehicle enterprises is uneven, and the competition in the industry is also continuously intensifying [4]. For enterprises to successfully transform and upgrade, they must improve technological levels, product quality, production efficiency, and scale capabilities through technological innovation and enterprise management, adopt advanced management models, shorten delivery cycles, reduce production costs, and enhance market share with good differentiated services.

For automotive companies to stand out in fierce competition and enhance their competitiveness, it is essential to study and improve new production processes. In line with the requirements of lean production, they must identify and resolve bottlenecks in production and the resulting waste of personnel, technology, equipment, and other issues. This will enable process reengineering and continuous innovation in production management models, optimizing production efficiency and costs control to achieve high-quality development. The workshop production line is the venue for manufacturing enterprises to carry out actual production activities and create wealth. Effective production system management can ensure excellent performance in terms of safety, quality, efficiency, and costs in workshop operations [5]. The internal wealth-creating activities of the company are all completed on the workshop production line. Based on a deep understanding of the current standard production process methods of the workshop production line, analyzing the characteristics of the current process steps and key stages of the production process, as well as the deficiencies and issues that need to be improved in the production phase, guided by the concept of lean production, a standard lean production management system is created. Continuously discovering and eliminating various forms of waste and irrational conditions in the production activities during subsequent production practice activities, thereby summarizing the best production process plan for the current production status of the automotive company’s painting workshop.

The painting workshop, as a key and important link in the automobile production process, could ensure the safe production plan of vehicles with the existing production line process when electric vehicles had not yet emerged. However, with the increasing order information in the workshop and the intensification of market competition, the original extensive production management mechanism of the workshop has become relatively outdated [6]. There is a serious waste of resources, the production process is backward, and issues such as delayed communication between departments, low staff quality, and changes in organizational patterns have led to increased production costs, low efficiency, and a significant decline in company profits. At the same time, the production characteristics of the automotive industry have also undergone a clear transformation, shifting from the previous few varieties and mass production to multi-variety, small batch production or multi-variety, multi-batch production. Product development and production must also meet various environmental protection and energy consumption indicators. The previous high-pollution, high-energy consumption production methods must transition to green, low-energy consumption. Companies are under increasing pressure. To enhance the company’s existing status, lean management should be used to support the optimization and reconfiguration of the whole manufacturing process, continuously promote the improvement of the professional capabilities of all personnel, carry out standardized and optimized management for each work link, improve production efficiency and product quality, ensure energy saving and consumption reduction, eliminate consumption and environmental pollution throughout the entire production process, and improve and optimize the production system software of the enterprise, thereby enhancing the company’s comprehensive market competitiveness.

Digital technologies, including communication technologies related to mobile communications and the Internet of Things, cloud computing technologies related to high-performance computing and storage, artificial intelligence technologies related to automatic reasoning, search, and machine learning, and big data technologies related to data mining and analysis, are leading the society and economy of the digital era [7]. Science and technology advancements have accelerated the growth of conventional sectors, and digital transformation is rapidly becoming a strategic emphasis for achieving competitive and long-term economic benefits [8]. Digital transformation is the process of altering companies and organizations’ key business processes utilizing digital technology tools and concepts, resulting in the move from traditional business models to digital transformation. Digital transformation entails not only technological upgrades and updates, but also changes in all elements of enterprise leadership and structure. In China, various forms of policy advice and assistance have been actively implemented, with the ongoing publication of plans for the in-depth integration of digitization and industrialization, strengthening the digital application of manufacturing, breaking through key common technologies for reducing carbon emissions in manufacturing, and promoting the high-quality development of manufacturing [8]. Digital transformation endows physical enterprises with new development momentum, which can optimize internal control, improve organizational processes and structures [9]; Porfmo et al. [10] believe that in the process of digital transformation, if the management supports the enterprise’s digital transformation and implements digital management using digital technology, it can accelerate the development process of the enterprise; in the era of big data, small and medium-sized enterprises continuously and effectively combine business and digital strategy, help build the enterprise’s digital network by collecting and organizing various information resources, respond quickly to external changes, serve corporate strategic decisions, and jointly promote enterprise development [11]. Manufacturing also faces substantial cost constraints as it develops digital technology [12].

China is particularly suitable for the study of lean production and digital transformation in electric vehicles. In September 2020, China announced to the world at the United Nations General Assembly its goals to achieve carbon peak by 2030 and carbon neutrality by 2060. The proposal of the “dual carbon” targets not only responds to the Paris Agreement and actively addresses climate change, demonstrating the responsibility and commitment of a major country, but also has a far-sighted strategic significance in accelerating the economic and energy transformation of China [13]. China produces and sells more than 60% of the world’s electric cars and has been rated number one for nine years in a row [14]. As the world’s second-largest economy and the largest manufacturer of electric vehicles globally, China’s digital development is immense, and the study of the link between the growth of electric cars in China, lean manufacturing, and digital transformation has crucial guiding implications for emerging countries.

The rest of the article is organized below. The introduction section 1 outlines the research background, purpose, and significance, section 2 is a literature review, section 3 describes the workshop’s current situation, data sources, and data analysis results, section 4 reports the empirical analysis results of VSM technology based on the digital transformation environment, and section 5 presents the conclusions, managerial implications and future research prospects of this study.

## 2. Literature review

In order to improve efficiency and management levels, enterprises have been transitioning from traditional production methods to lean production methods. The application of VSM as an effective tool for implementing lean production has yielded good value-added results. In 1988, Krafcik, a member of the International Motor Vehicle Program at the Massachusetts Institute of Technology, first introduced the concept of the lean production system in his article “Triumph of the Lean Production System” [15]. By comparing the production efficiency of automobile manufacturing enterprises in Japan, Europe, and the United States at that time, he found that the production systems in these countries were broadly divided into two types: buffered and lean. In 2004, Professor Liker from the University of Michigan, starting from the ideas and culture of Toyota’s creation of the Toyota Production System, revealed that the main notion of the Toyota Production System is “elimination of waste” and “explained the operability of the Toyota model with 14 principles,” proposing theories and methods for transforming enterprises based on the Toyota Production System [16]. Falsafi pointed out that in lean production, the inbound logistics connect suppliers with manufacturing enterprises to ensure the stability and continuity of subsequent production [17]. Marcos et al. have pointed out that lean management has been developed and extended to the technology industry, providing efficiency benefits for production operations in the shipbuilding and aerospace industries, such as reduced wait time, processing duration, duration of cycle, installation time, levels of inventory, defects, and waste, as well as improvements in overall equipment efficiency [18]. Zhang et al. [19] took the Qingdao Kute Company as the research subject, enriched the theoretical research of data empowerment and lean production, and also had practical significance in guiding enterprises to create data-driven lean production advantages. Wang et al. [20] took the A workshop connector assembly process as an example, studied the improvement of the connector assembly process using lean tools and achieved good results. Some enterprises in China have not achieved good results in implementing lean production for two main reasons: on the one hand, most enterprises have not understood the connotation and core of lean production, focusing only on the use of lean production tools, with limited and unsustainable improvement effects; on the other hand, when enterprises hire lean experts and managers who have successfully implemented lean from outside, the goals they set are often too high, without addressing the real condition of the firm, resulting in the abandoning of lean manufacturing halfway [21].

VSM technology was proposed by Mike Rother and John Shook in June 1988, based on their study of Toyota’s experience [22]. In the past few years, academics at both domestic and international levels have undertaken considerable study on lean manufacturing and the use of VSM technology in different production processes of firms, obtaining good production gains [23–26]. Zhou et al. explored the lean approaches of VSM in the production line of complex electronic products. By analyzing the production process, they constructed the existing Value Stream Map. Through data analysis, they identified non-value-added time and, by introducing lean thinking and methods such as production line balancing, reduced non-value-added time, shortened product cycles, reduced production costs, and increased enterprise benefits. Shi introduced lean thinking and transformed the production line process from three aspects: production cycle, product quality, and inventory volume; thereby, the industrial process’s value stream is constantly improved. Masuti et al. proposed that the use of Value Stream technology in an excavator manufacturing company achieved the goal of lean production, and Munyai et al. improved production efficiency in a steel shaft manufacturing setting by simulating the application of Value Stream technology to actual production.

Michael Hammer of the Massachusetts Institute of Technology published an article in the Harvard Business Review in 1990, proposing that “business process reengineering is to fundamentally consider and thoroughly design business processes so that they achieve significant improvements in indicators such as cost, quality, service, and speed” [27]. This theory has been widely studied in various industries abroad [28–31], for example, Johnson & Johnson and Ford Motor Company have reaped substantial benefits from business process reengineering.

Based on the theory of lean production, combined with the ECRS optimization principles, the enhancement of corporate competitiveness is achieved by reducing waste, improving efficiency, and increasing customer value [32]. E (Eliminate) refers to the cancellation of unnecessary processes, operations, actions, investments, etc. Elimination is the best effect and the highest principle of improvement. C (Combine) refers to the merging of necessary elements that cannot be eliminated, which can be combined according to the actual situation to achieve the purpose of saving time and simplification. R (Rearrange) refers to the reordering of tasks that cannot be eliminated or combined to optimize the task sequence. S (Simple) means simplification, which means considering the most simple and optimal method to complete the work after elimination, combination, and rearrangement. Liu [33] applied the ECRS concept to analyze the actions and time of the bridge spherical bearing production line, achieving line balance of various operation units of the bridge spherical bearing by eliminating non-value-added operations and optimizing the reorganization of work content. Smith et al. [34] studied the performance of each element in the human-robot collaborative system based on the cost function, evaluated the time and fatigue level of workers completing assembly tasks, and optimized the process flow. Fang et al. incorporated the enterprise’s product production quantity, product transportation and distribution, and product market pricing into a single system, constructing an integrated decision-making model, so that when pricing products, full consideration can be given to the production cost, transportation cost, and market demand of the enterprise’s products [35]. Li et al. analyzed methods and cases of automotive manufacturing process optimization and lean manufacturing management, dissected the PDCA method continuous improvement cycle mechanism, thereby promoting lean production and cost reduction, and constantly upgrading the quality of automobile products to respond to the increasingly competitive marketplace environment [36]. Liu et al. coordinated the single workshop production and various modes of synchronous transportation in the order production process of enterprises, further shortening the completion time of enterprise orders [37]. Literature [38] proposed an improvement plan for the problems in FH Company’s new product development project management process, improved the process, restructured the project management team’s organizational structure and responsibility matrix, improved customer satisfaction, and increased team efficiency. Literature [39] pointed out that in the process of process reengineering, it is necessary to sort out and improve the processes of related supporting businesses, promote the entire reengineering project from the enterprise level, organize the whole process, and achieve the transformation of optimized processes, thereby improving quality, efficiency, and benefits.

The integration of digital technology and smart terminals strongly promotes the development of the digital economy in various industries. In terms of health, through the computational modeling of public health and medical health data, the active prevention of diseases is realized; at the same time, with the help of IoT, AI, and other technologies, the treatment plan will no longer be the same. In terms of diet, artificial food that meets individual health needs and is rich in flavor will be obtained through 3D printing technology to meet human nutritional needs. In terms of housing, based on the next-generation Internet of Things operating system, to realize the self-adaptation of home and office environments so that people have a space that “understands you.” In terms of traveling, new energy vehicles with automatic driving technology will allow us to have an exclusive mobile third space; new manned vehicles will not only enhance the efficiency of emergency rescue and reduce the cost of transporting emergency supplies, but will also change the way people commute.

With the swift growth of computer information technology, Tapscott’s “Digital Economy Theory” has extended the notion of the digital economy to the era of networked intelligence. This information system is not limited to the integration of digital technology and intelligent terminals; it refers more to the environment of production and life between people based on technology [40]. The digital economy has developed along with the formation and growth of digital information in this century. It not only encompasses traditional fundamental fields like electronic communication and the electronic information industry, which are collectively called the digital information industry, but its scope has also been further broadened. It has begun to emerge as a collective word for economic sectors that include industrial digitalization, informatization, and intelligence, as well as an economic model that has evolved and expanded by incorporating digital information into numerous sectors. Nowadays, various industries attach increasing importance to the development of the digital economy, gradually transitioning from the traditional economy to the digital economy, giving external support to the transformation and development of the industry; The manufacturing industry is also continuing to increase its attention to the digital economy, helping the manufacturing industry move toward a higher level of information and intelligence, and injecting internal driving forces into the development of the manufacturing industry with digital transformation [41]. Many benefits have resulted from digital transformation, including the simplification of corporate processes, increased operational efficiency, integration of internal and external resources, promotion of company structure innovation, and promotion of manufacturing system upgrades. By utilizing digital technology and continual creativity, the technological and managerial level of firms may be actively upgraded, considerably boosting the inventive potential of the manufacturing sector and fostering its sustainable growth.

The high value-added ratio and production efficiency of the electric vehicle body painting production line are achieved through the application of technological innovation such as value stream analysis of lean production and digital transformation. Compared to existing literature, this paper contributes in the following ways: Firstly, it takes lean manufacturing as the entry point, enriching the research on the optimization of automotive production lines based on lean manufacturing theory, and showing the economic and environmental benefits of lean manufacturing for NEV development. Secondly, through the study of VSM technology, it extends and verifies the favorable influence of VSM technology on improving the productivity of NEV production lines. Thirdly, it focuses on the impact of digital transformation on improving the quality of automotive painting processes, which is beneficial to the long-term growth of the automobile manufacturing sector and advances China’s dual carbon targets. Fourthly, this research helps to broaden the horizons of literature on VSM technology and lean management of NEV production lines under the backdrop of digital transformation.

Lean VSM techniques have been used in many industries but not much in the field of automotive production body painting lines. Murat et al. Reference [42] presented a case study on the deployment of an RFID-based electronic kanban system in a supplier company in the automotive industry, generating current value stream maps and future value stream maps, establishing performance metrics, and conducting a benefit-cost analysis to reduce inventory levels within the production system. Ribeiro et al. [43] implemented lean tools in automotive products such as wheel covers and front bumpers, which resulted in shorter cycle times, increased production line yields, and a reduction in complaints related to these products. Improvements were suggested through lean tools and methods (e.g., 5S, VSM), which resulted in a 16% increase in the OEE index of the wheel cover painting line and a 17% increase in the OEE index of the painting line for the front bumper. No previous body painting line study has considered the case of combining digital transformation and VSM lean management methodology, which is of great theoretical and practical significance in today’s rapidly developing digital economy.

This paper studies the new energy automobile production line based on technological transformation and through the value stream mapping technology of lean manufacturing and digital transformation to realize the new energy automobile body coating technology level of high value-added ratio and production efficiency. Compared with the existing literature, this paper has the following contributions: first, taking lean manufacturing as the entry point, it enriches the research on the optimization of automobile production line based on the theory of lean manufacturing, and demonstrates the economic and environmental value of lean manufacturing in the development of new energy automobiles; second, it extends and verifies the favorable impact of value stream mapping technology on the efficiency improvement of new energy automobile body painting production line through the study of value stream mapping technology; third, focusing on the impact of digital transformation on improving the quality of automotive spraying process, which is favorable to the sustainable development of the automotive manufacturing industry and promotes the realization of China’s dual-carbon goals; fourth, this study helps to broaden the horizons of the literature on value stream mapping technology and lean management of new energy automotive body painting production lines in the environment of digital transformation.

## 3. Analysis of the current situation of the painting workshop based on VSM technology

Taking the painting workshop as the research subject, using the opportunity of enterprise practice activities to grasp the first-hand data and information on the production process management methods of the TJ production workshop, and to analyze and judge the effects of all new production processes at any time. The scientific research on the optimization of the production process in the TJ enterprise workshop mentioned in the paper can further enrich and improve the research theory of Chinese-related electric vehicle production processes and lean production, enhance the management level of enterprises, and increase their economic and social benefits. At the same time, it provides successful case references for the application of production processes and lean production for Chinese automotive manufacturing enterprises, thereby continuously promoting the excellent development of the automotive manufacturing sector.

### 3.1. Analysis of the current production situation

TJ Automobile Manufacturing Plant is a designated national production enterprise for light commercial vehicles and special modified vehicles. It has more than 20 domestic advanced automobile production lines including stamping, welding, and testing, and has formed an annual production capacity of 100,000 complete vehicles. Since domestic and international research on lean production value stream is mainly focused on the assembly manufacturing process, and the automotive painting, which is a manufacturing system with a relatively substantial level of automation, is quite special, there is less research in this area. Therefore, the car body painting production system is chosen as an example for analysis and research.

The painting workshop includes four processes: car body painting, plastic parts painting, cargo box painting, and frame painting. Due to the complexity and high requirements of the car body painting process, the study focuses on this particular procedure. The process consists of pre-treatment, electrophoretic drying, sealing and glue injection, glue drying, electrophoretic grinding, painting, paint drying, and finishing, culminating in the handover. It is divided into 8 major operations with a total time of 221 minutes and involves 16 workers. S1 Table presents the production operations, working time, and number of workers in the car body painting workshop. The production method adopts batch production and is designed according to three shifts of continuous cycle work, with a line body painting output of 10,000 sets/year.

In addition to value-added time, there are also non-value-added times such as preheating, material collection, fixing, and paint mixing, as shown in S1 Fig. By analyzing the time allocation of the painting production process, it is found that the allocation of resources and working hours on each process is unbalanced, with long waiting times, leading to waste. Taking the painting process as an example, since the drying time of the painting process must be guaranteed for 32 minutes, the data analysis from S3 Fig shows that there are 3 processes in the painting production process that exceed 32 minutes. If the time of these 3 processes can be improved by compressing the working time within 32 minutes, it would ensure a balanced allocation of working hours for each process and reduce waste in the processing.

### 3.2. Current situation analysis of VSM

Value stream analysis is at the core of lean production philosophy and is essential in transforming production methods and processes. It involves identifying the production segments that generate value and using these as a foundation to pursue the maximization of benefits. The ultimate outcome of value stream analysis is to propose and implement improvement plans for value-adding production segments, followed by their evaluation of effectiveness. S2 Fig shows the process of value stream analysis.

TJ Automobile Manufacturing Plant’s car body painting workshop operates from Monday to Saturday, with one shift per day, and each shift works for 8 hours. S3 Fig depicts the current value stream map of the vehicle body painting production process, which is based on the order procedure, manufacturing technology, and basic data.

## 4. Research on painting process management based on VSM technology in the context of digital transformation

### 4.1. ECRS process optimization waste analysis

ECRS is an important optimization principle in lean production, which enhances processes and work methods by eliminating, combining, rearranging, and simplifying through four steps, thereby improving efficiency and reducing costs.

Lean production defines potential waste in the process as any action that uses energy without providing benefit to the consumer. The most significant wastes are categorized into seven types: waiting, transportation, over-processing, unnecessary motion, defects, overproduction, and inventory waste. By applying the ECRS optimization principle of lean production to the current production status of TJ Automobile Manufacturing Co., Ltd., a careful optimization study of the company’s car body painting production system has been conducted to identify seven issues that significantly impact the value-added ratio of the car body painting production system, and analyze the causes of each of the seven problems and give solutions to the problems from the four optimization principles, so as to reduce the waste and improve the production efficiency. As shown in S2 Table.

By analyzing the current situation through VSM and in conjunction with the actual production situation, a lean production approach is used to analyze the current state of the TJ Automobile’s car body painting production system’s value stream. It is found that there are mainly the following potential waste issues affecting the system’s value-added ratio:

(1) The sealing and glue injection process has a long cycle time, with uneven workload between workstations;
(2) The spray painting process has a long cycle time, with lengthy process times and unstable quality;
(3) The finishing process is lengthy, increasing non-value-added time;
(4) The processes of paint mixing and spray painting are non-value-added and wasteful, with long cycle times;
(5) The spray painting does not meet environmental standards, which is not conducive to environmental protection, preventing the company from starting production.

### 4.2. Research on improvement of VSM technology based on digital transformation

#### Formulation of improvement plan.

Through digital transformation analysis, we aim to address seven issues by fully upgrading and optimizing traditional business models, processes, and culture using digital technology and means. This involves multiple aspects, including technology, organization, and strategy, to help enterprises and organizations improve efficiency, create greater value, enhance competitiveness, and explore new business opportunities and growth potential. From the current state diagram, it is evident that TJ Automobile Manufacturing Plant’s painting workshop employs a push production planning mode, where each process starts only after the previous one is completed. To improve the VSM activities derived from the analysis, potential issues in each activity need to be analyzed and improvement plans formulated as follows:

***Optimization of sealing and gluing process:*** The sealing and gluing process has a long production cycle and many stations, resulting in a prolonged total system throughput time, which hinders effective output improvement. By conducting a stopwatch time study on the operation times at each station of the sealing and gluing line. S3 Table shows the results of the study.

The long operation times at each station affect the production cycle of the entire section. To improve this situation, we will enhance personnel digital skills and standard work training to proficiently use digital tools for quick door fixture changes, the cycle time and gluing process cycle time are reduced by 2 minutes and 1 minute respectively. In the repair glue process, we will adopt machine vision based on digital image processing to quickly determine the repair glue positions, reducing the cycle time by 0.5 minutes. After improvement, the total cycle time of the section is 29 minutes, a reduction of 5 minutes, keeping the total production process waiting time below 32 minutes and improving production efficiency.

***Optimization of painting process*:** The painting process, due to inefficient manual spraying operations and long production cycles, has many stations, resulting in a prolonged total system throughput time, which hinders effective output improvement. By conducting a stopwatch time study on the operation times at each station of the painting line. S4 Table shows the results of the study.

Among the six painting stations, except for a few manual touch-up stations, the manual spraying stations will be eliminated, and four automatic spraying robots will be added for automated spraying. The flexible configuration of the spraying robots allows for multi-directional spraying, reducing each automatic spraying cycle time by 2 minutes. Through personnel skills and standard work training, and the utilization of robots, each manual spraying cycle time is also reduced by 2 minutes. After improvement, the total cycle time of the section is 30 minutes, a reduction of 12 minutes, keeping the total production process waiting time below 32 minutes, and the personnel are optimized to 2 people, improving production efficiency. Additionally, due to the digital spraying robots’ dual functions of offline programming and point-to-point programming, they can flexibly adjust the motion of each component during spraying without reprogramming, providing uniform and standard film thickness spraying quality, thereby improving product quality with the optimized process.

***Optimization of finishing operation time*:** In this process, the surface defects of the workpiece paint are repaired through marking, grinding, and defect polishing. Analysis reveals that the finishing process takes 25 minutes with three operators, as shown in S5 Table.

Field process observation and investigation show that due to unstable manual surface spraying quality, employees spend most of their time repeatedly checking for defects, creating a high workload “illusion” but actually resulting in significant waste. After configuring action measures such as digital paint mixing and automatic spraying by robots, the quality of top coating and spraying has been improved, and the time for sanding and polishing has been reduced. Through digital monitoring and inspection technology, the quality is continuously monitored and inspected, and a machine vision recognition system is configured to automatically identify defects, which overcomes the shortcomings of slow manual visual inspection, susceptibility to fatigue and emotions, shortens the inspection time, improves the inspection accuracy, and realizes the effect of quality stability control. In this way, through on-site time measurement and verification, the whole section beat time can be set at 21 minutes, a reduction of 4 minutes, and staff optimization to 2 people. This optimization achieves the effect of increasing the effective output and reducing the operating costs.

***Optimization of non-value-added processes*:** The intermediate paint mixing process is a non-value-added process with long waiting times, resulting in a prolonged total system throughput time, which hinders effective output improvement. Additionally, due to manual paint mixing difficulties in color matching, it adversely affects the painting quality. Analysis reveals that the paint mixing process is a non-value-added process taking 42 minutes with four operators, as shown in S6 Table.

Traditional paint mixing mode due to long time and poor quality, naturally at a disadvantage, relying on manual experience and management experience, heavy pollution of the operating environment, high risk, serious staff turnover, paint workers difficult to start, low efficiency, high management and training costs, paint mixing process quality is not controllable, out of the problem of difficult to trace, and so on. The purpose of digital paint mixing is to achieve an intelligent, visual, and accurate ratio, breaking the limitations of experience, fast paint, color accuracy, material information from the screening, refusing to waste raw materials, and paint mixing. The whole process can be traced back to facilitate the manufacturer’s self-checking, to solve the existence of a manual process, inefficiency, poor color quality accuracy, and other issues, and cost savings at the same time save time to mix the paint. With the use of painting robots, an existing product color database can be established using industrial control computers. When color information is assigned to the vehicle, the required product color can be quickly exported through the human-machine interface, achieving rapid production changeover, and increasing the productivity of the spraying process and the entire production line. The use of painting robots reduces worker position change time, improving production efficiency and quality. The non-value-added process time in painting is reduced by a total of 34 minutes. Digital transformation can prompt enterprises to shift from “business-driven” to “data-driven,” aiding decision-makers in comprehensively understanding the enterprise status, conducting intelligent decision analysis, thereby enhancing decision-making ability and forming strong market competitiveness.

***Intelligent control of green painting waste gas treatment technology*:** Automobile touch-up and painting production lines are among the stages in the automotive industry that generate the most “three wastes,” with painting organic waste gas being a key part of painting environmental pollution. Organic waste gas in the paint shop mainly comes from solvents contained in the paint and dissolved substances during the entire paint drying process, commonly referred to as volatile organic compounds (VOCS). The key components are toluene and xylene, which are harmful to health and the environment. As environmental protection emission requirements become stricter, non-compliant emissions will prevent enterprises from commencing production.

An integrated digital control system has been developed, utilizing IoT technology integrated with smart hardware to first achieve VOCS gas detection. If non-compliance is detected, the gas purification device is activated, enabling continuous zeolite rotor adsorption. The concentrated VOCS gas is then subjected to high-temperature combustion treatment via a TNV recovery-type thermal oxidation system (or other equipment). The generated heat is used for heating the drying chamber and zeolite rotor desorption, making full use of the heat, achieving energy saving and emission reduction effects.

Automobile production spraying link, according to the “Air Pollution Prevention and Control Law” “Comprehensive Emission Standards for Air Pollutants” and the local stricter standards for VOCS concentration of car companies paint booth concentration purification treatment emission concentration of ≤  20 mg/m3. Digital control system according to the “Comprehensive Emission Standards for Air Pollutants” and “Determination of Volatile Organic Compounds of Fixed Pollutant Sources of Exhaust Gases” the national standard set parameters for detection, calculation of the appropriate The VOCS emission concentration value of the corresponding section is calculated, and after the detection does not meet the standard, the gas purification device is opened for treatment until it meets the standard requirements. Intelligent control of green coating exhaust gas treatment technology spraying production line due to the use of composite treatment technology, exhaust gas purification rate from the original 32% to 98%, exhaust emissions from the original 30 mg/m3, reduced to 9 mg/m3, far below the national standard.

#### Drawing the future VSM.

By analyzing and utilizing the above VSM technologies, a digital transformation-based body coating process route management method was tailored to address the challenges in the body coating process at TJ Automotive. Through the skilled use of door support quick-change digital tools and the use of machine vision to automatically identify defects, the sealing injection process time has been optimized; through the use of digital automatic spraying robots for intelligent spraying, the paint spraying process time has been effectively shortened; through the digital monitoring and inspection technology, the quality of continuous monitoring and inspection has been shortened; through the digital paint mixing has been realized intelligently, solving the problems of manual processes, efficiency, color, and color. The existence of manual processes, low efficiency, poor color quality accuracy, and other issues; saving costs while saving paint mixing time; increased intelligent control of green painting exhaust gas treatment technology; to achieve the requirements of environmental protection. Overall, the value-added operation time increases by 21 minutes and the non-value-added operation time decreases by 34 minutes, which effectively improves the production efficiency of body painting.

Based on the proposed improvement plans for each activity above, to achieve improvement goals and endpoints, and to lay the groundwork for the next cycle. As shown in S4 Fig, a future value stream map is plotted.

### 4.3. Improvement benefit analysis

#### Establishment of lean improvement evaluation system.

By implementing lean production improvements based on digital transformation on the production line, all processes of the body painting production line have gradually reached a balance, and on-site arrangements are becoming increasingly reasonable. To evaluate the performance of lean production improvements, a scientific evaluation system must be established. Through scientific evaluation, one can clearly understand the achievements and areas for improvement in implementing lean production, which will guide the development of the enterprise, enhance the confidence in continuous improvement, and serve as a demonstration to help other companies recognize the value of lean production.

Adopting the method of data statistics and collecting information about lean production improvement according to the characteristics of the automobile manufacturing industry, combined with the enterprise’s own production characteristics, a set of painting production process lean production evaluation systems was established, with the following specific improvement objectives and analysis and calculation methods:

1. Value added in the production process

Based on the value stream map analysis, the value-added work time in the car body painting production process is:


$$VAT=\sum_{i=1}^{n}T_{i}$$

where $T_{i}$ represents the value-added time for the i-th operation.

Non-value-added work time:


$$NVAT=\sum_{i=1}^{n}T_{i}^{\text{‘}}$$

Value added operating time increase ratio (VATIR):


$$VATIR=\left(VAT1-VAT2\right)/VAT1$$ (1)

Where VAT1 is the initial value-added operating time.

VAT2 is the improved value-added operating time.

Non-value-added operating time reduction rate (NVATRR):


$$NVATRR=\left(NVAT1-NVAT2\right)/NVAT1$$ (2)

Where NVAT1 is the initial non-value-added operating time.

NVAT2 is the improved non-value-added operating time.

Where $T_{i}^{\text{‘}}$ is the non-value-added work time for the i-th operation.

Value-added ratio (VAR) is:


VAR=VAT/（VAT+NVAT） (3)

1. Improvement of production rate (IPR)

$$IPR=\left(PM2-PM1\right)/PM1$$ (4)

Where PM1 is the original number of pieces produced per month.

PM2 is the current monthly production number.

1. Production personnel reduction rate (PRR)

$$PRR=\left(PP1-PP2\right)/PP1$$ (5)

Where PP1 is the original number of production personnel.

PP2 is the number of production personnel.

1. Improvement of production quality (IPQ)

IPQ=RR2−RR1 (6)

Where RR1 is the original yield rate and RR2 is the current yield rate.

1. Improvement of exhaust gas purification rate (IGPR)

IGPR=EGPR2−EGPR1 (7)

EGPR1 is the original exhaust gas purification rate.

EGPR2 is the current exhaust gas purification rate.

#### Analysis of improvement results.

Through the data statistics, the original and present actual production data of the body painting system before and after lean production are listed, and the effect of lean production improvement can be calculated based on equations (1)-(7), as shown in S7 Table.

Based on the data analysis shown in S7 Table, quantitative evaluation and qualitative summary of various improvement plans were conducted. The specific improvement effects are as follows:

***Reduction of total system working time*:** By optimizing the resource allocation for every process, the system’s total time at work was lowered. By optimizing the time allocation of each process and ensuring the 32-minute drying time for the coating process, the time for three processes exceeding 32 minutes was reduced to within 32 minutes, ensuring balanced work time allocation for each process, reducing long waiting times and waste. As shown in S5 Fig, the cycle time of the sealing and gluing process was reduced by 5 minutes, the painting process was optimized to reduce the cycle time by 12 minutes, the fine finishing operation time was reduced by 4 minutes, and the non-value-added time for intermediate mixing and painting was reduced by 34 minutes, reducing the total working time of the body painting system by 53 minutes, improving the system’s effective output.

***Improvement in system value-added rate*:** Through various improvement activities, the value-added operation time in the production process increased by 9.5%, and the non-value-added operation time decreased by 12.5%, improving the value-added ratio of the future VSM.

***Reduction of operating costs*:** Two workstations were eliminated in the painting process, and one workstation was eliminated in the fine finishing process, reducing the number of operators by three and decreasing personnel by 18.8%.

***Improvement in product quality*:** The application of robotic painting production lines and digital machine vision inspection systems improved production efficiency and product quality, reducing the defect rate by 15%, which helps to improve the production and sales rate of the system and increase the company’s profit.

***Improvement of the atmospheric environment*:** Intelligent control green painting exhaust gas treatment technology effectively reduced VOCS emissions, increased the exhaust gas purification rate by 66%, and fully utilized the heat of the exhaust gas, achieving energy-saving and emission reduction effects.

## 5. Conclusions and insights

### 5.1. Conclusions

(1) Taking the electric vehicle body painting production system of LT company as an example, a new method of process reengineering by combining VSM analysis technology and process reengineering ECRS optimization principles with lean production theory was proposed, analyzing the problems in the current production status, and identifying waste in the automotive body painting production system, laying the foundation for process reengineering improvement plans.
(2) Application of digital transformation a new method of applying VSM analysis technology in the context of digital transformation is proposed. The electric car body painting production system’s process plan was improved by utilizing digital technology, organization, and strategy, increasing the value-added operation time of the painting production process by 9.5%, reducing the non-value-added operation time by 12.5%, enhancing the system value-added ratio, reducing the production cycle by 53 minutes, and decreasing personnel by three, thereby improving the system’s effective output.
(3) Through the application of digital robot painting technology and optimization as well as improvement of the production process, the quality of body painting products is improved, the defect rate is reduced by 15%, operating costs and inventory are lowered, and the exhaust gas purification rate is increased by 66%, reducing environmental pollution. This provides a successful case for the upgrading and transformation of the electric vehicle painting system, with significant implications for the sustainable development of the automotive manufacturing industry.

### 5.2. Research insights

From a theoretical point of view, economic development in the digital economy prioritizes both high economic advantages and long-term development. Lean production and digital transformation are current research hotspots. Based on synthesizing and applying existing study findings, this paper further enriches the theoretical level of combining VSM analysis technology and process reengineering ECRS optimization principles with lean production theory and applying new methods of VSM analysis technology in the context of digital transformation. Selecting recent data from the electric vehicle manufacturing painting system for empirical analysis, with scientifically reasonable data selection and strong timeliness, comprehensively exploring the production and management issues of the automotive manufacturing painting system from three aspects: VSM analysis technology, ECRS optimization principles, and digital transformation applications, avoiding the limitations of single-factor research. Through the integration of relevant theories and literature, this paper expands and improves the knowledge system of this subject and provides a new research perspective for this field. As the automotive industry is a branch of the manufacturing industry, this paper suggests that the method is also applicable to the automotive industry outside of other manufacturing processes, such as robotics, machine vision inspection technology, intelligent control technology, etc., but in the specific application of the other manufacturing processes, to be adjusted for the different specific circumstances necessary to adapt to different manufacturing processes. Such as precision manufacturing environments to consider more stringent environmental protection and high precision requirements; high-volume product manufacturing industrial environments to consider improving the level of automation and the level of intelligent control systems.

From a practical perspective, compared with developed countries, China’s automotive manufacturing industry started relatively late. Although the development trend of electric vehicles has been significant in recent years, many automotive manufacturing-related enterprises still face development difficulties. Based on this study, the recommendations made in the summary are as follows:

#### For enterprises.

It is essential to promote lean production management throughout the entire enterprise and integrate lean production management into the company culture. Every employee needs to establish the concept of lean production management and diligently apply lean production management methods. A continuous improvement lean management philosophy must be created to ensure closed-loop management, along with establishing and improving related management systems such as inspection, evaluation, and continual enhancement are examples of ways to ensure that lean production management runs well. With the advancement of high-end equipment manufacturing, the continuous development of industrial robots and intelligent system equipment, and the rapid development of information detection and management technologies, future lean production management research will ultimately integrate further with intelligence and informatization. Under the general trend of China’s 2025 manufacturing plan and the international Industry 4.0, related industries in the manufacturing sector will eventually extensively use intelligent manufacturing systems. The large-scale application of intelligent manufacturing systems in the production process will gradually enhance the professional skills of employees in this field, ultimately sparking a new wave of lean improvement.

The automobile manufacturing industry should formulate a digital transformation development strategy to promote the rapid development of enterprise digital transformation and improve enterprise efficiency and innovation. First of all, enterprises should implement digital transformation with the help of digital technology to significantly improve the efficiency in production, operation, service, and other processes, optimize the business process, improve governance to improve the business organizational structure, and effectively reduce the cost of the enterprise. Secondly, with the help of digital transformation, constantly enhance the teamwork of the company’s employees, effectively saving the team personnel in the innovation process of research and development time; the use of big data for risk prediction, to further grasp the user’s needs, to reduce the uncertainty in the research and development process, and to reduce the risk of innovation. Furthermore, the popularization and use of information technology should be used to effectively improve the communication efficiency of the upper and lower industrial chains, open up the enterprise boundary barriers, reduce communication costs so that resources can be reasonably allocated, and greatly improve the efficiency of enterprise management. Finally, the use of digital transformation to efficiently integrate resources, promote the exchange and sharing of data and knowledge elements in the internal system of the enterprise, and help the enterprise with high-quality development.

The Chinese government has introduced various policies such as the Environmental Protection Law and the overall layout plan for Digital China construction. Manufacturing enterprises need to understand and respond to these policies promptly. Companies can retain more high-quality “brains” through training and introduction, promoting digital transformation across various functional departments such as procurement, production, marketing, finance, and human resources, accelerating the construction of digital infrastructure in the manufacturing industry. Employing digital technologies to enhance the total factor productivity of manufacturing, supervising information like raw materials and energy, and consumer demands via IoT and big data platforms, guaranteeing efficient resource allocation and unobstructed supply chains, further facilitating the high-quality development of the automotive manufacturing industry [44]. Reference [45] studies indicate that top-level digital technology managers can effectively guide their companies to cultivate a corporate culture, organizational structure, and management team that comply with the requirements of the digital age.

#### For the government.

It is necessary to strengthen financial and technical assistance for lean production and digital transformation initiatives in the automotive manufacturing industry. The government should fully recognize the importance of lean production and digital transformation as means to enhance the sustainable development performance of the automotive manufacturing industry. Policymakers should formulate effective policies to promote investments in lean production and digital transformation and provide targeted incentives. These policies and actions not only encourage the sustainable growth and resilience of the automotive manufacturing sector in the face of challenges such as the COVID-19 pandemic and global uncertainties but also ensure the long-term sustainability of the automotive manufacturing industry.

This study has several limitations. Firstly, it solely selects TJ Company in China’s car manufacturing business as the research object, with no consideration given to the application to other firms or comparison analyses. Secondly, this study is only applicable to the Chinese automotive manufacturing industry and may not be applicable to other countries. Moreover, this study focuses on VSM analysis technology, process reengineering, and digital transformation without considering other aspects, and does not conduct quantitative research on energy consumption and energy-saving issues. Future research can supplement relevant theoretical research from the above perspectives.

## Supporting information

S1 Fig Time allocation for each process in car body painting production. (TIF)

S2 Fig VSM analysis process. (TIF)

S3 Fig Current VSM of the car body painting production system. (TIF)

S4 Fig Future VSM of vehicle body painting production process. (TIF)

S5 Fig Optimization time configuration for various processes in car body painting production. (TIF)

S1 Table Car body painting production process, working time, and number of workers. (DOCX)

S2 Table Waste analysis in the painting production process. (DOCX)

S3 Table Operation time of sealing and gluing line before optimization. (DOCX)

S4 Table Operation time of sealing and gluing line before optimization. (DOCX)

S5 Table Operation time of finishing work before optimization. (DOCX)

S6 Table Non-value-added process time of painting operations before optimization. (DOCX)

S7 Table Improvement effects of TJ Company’s body painting production process. (DOCX)
