# Supplementary figures and images for: Study on lean production management of new energy vehicle body painting based on the dual perspectives of digital transformation and VSM
Source: PLoS One. 2025 Feb 14;20(2):e0318253. doi: 10.1371/journal.pone.0318253 (PMC11828361; doi:10.1371/journal.pone.0318253)

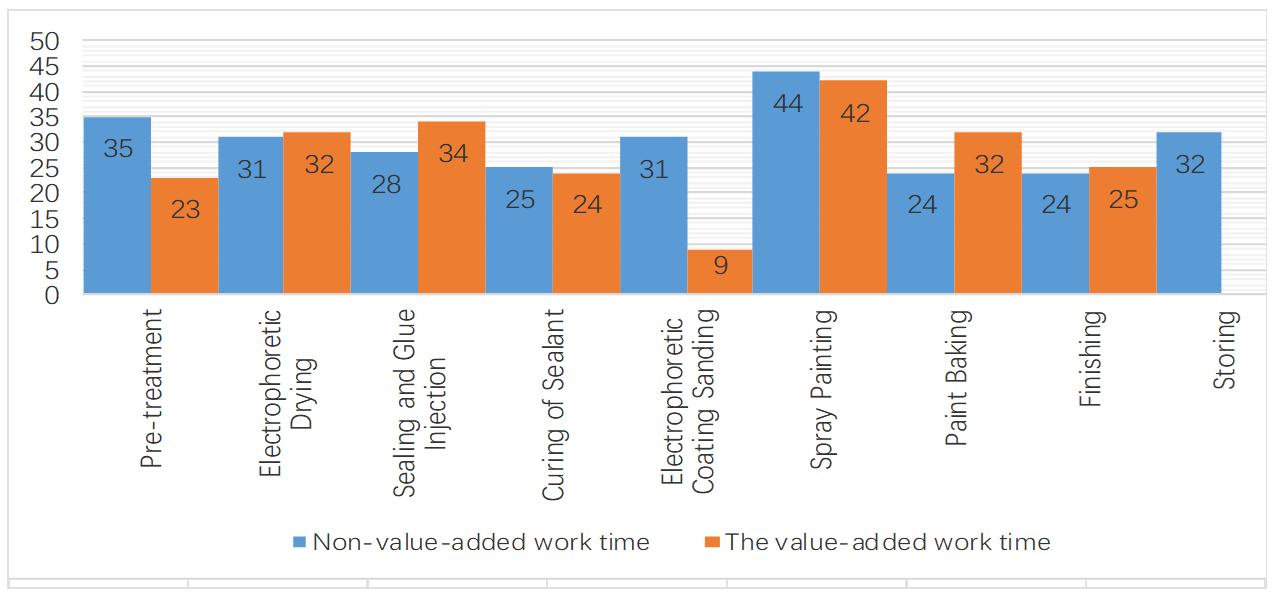

Supplement: S1 Fig — (TIF) [file pone.0318253.s001.tif]

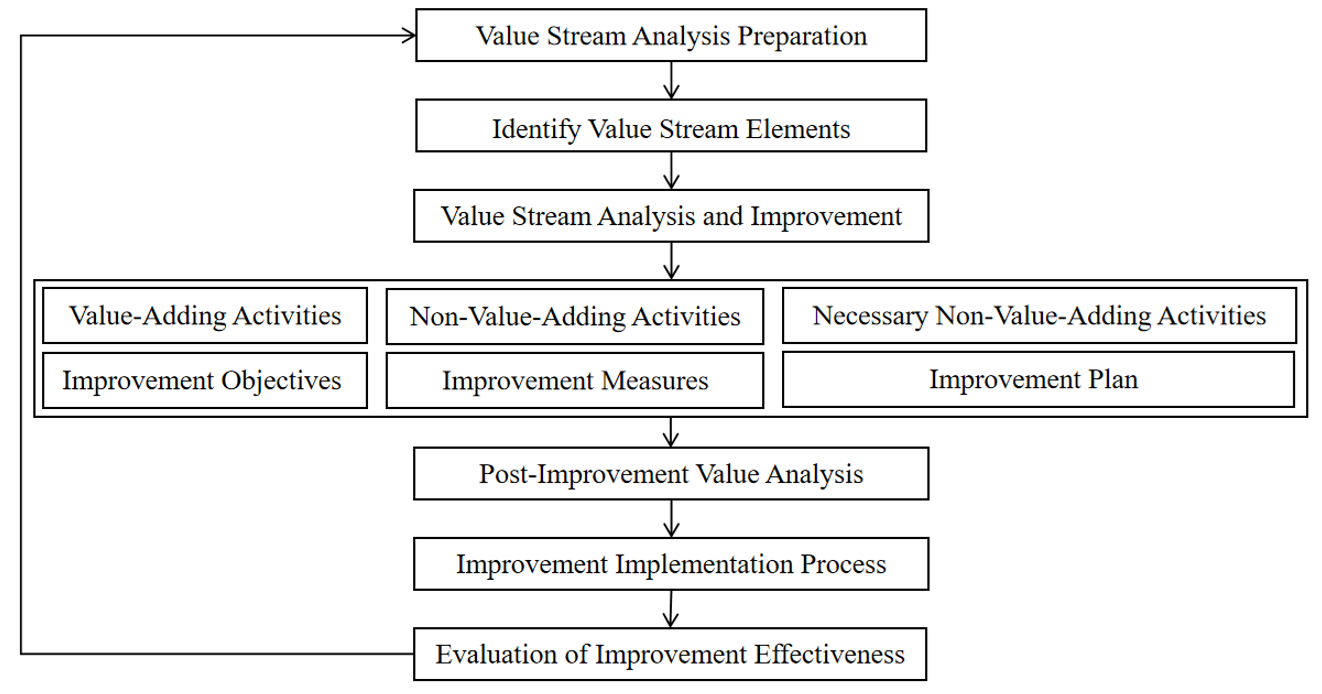

Supplement: S2 Fig — (TIF) [file pone.0318253.s002.tif]

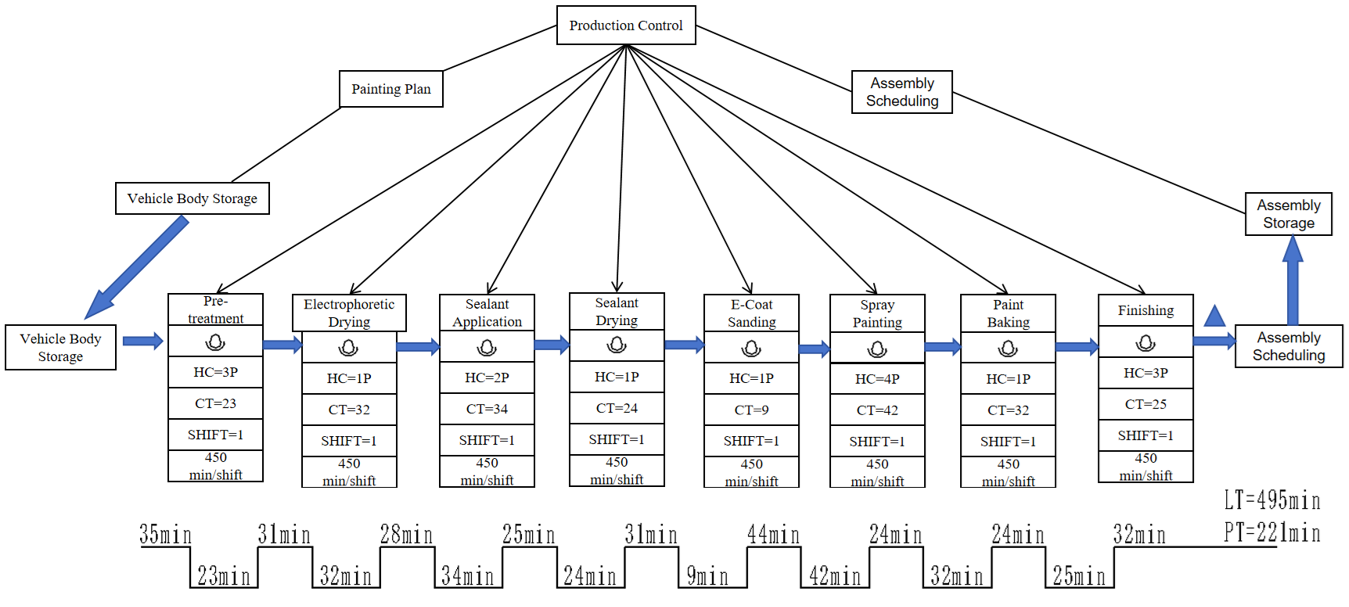

Supplement: S3 Fig — (TIF) [file pone.0318253.s003.tif]

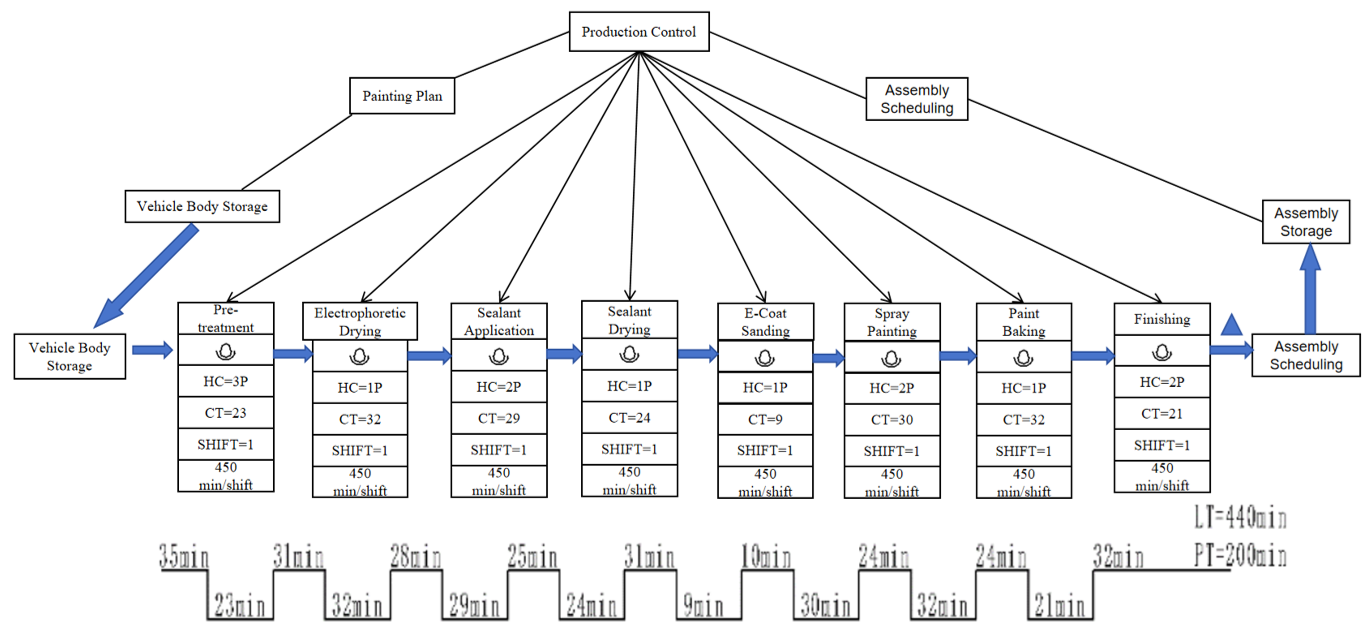

Supplement: S4 Fig — (TIF) [file pone.0318253.s004.tif]

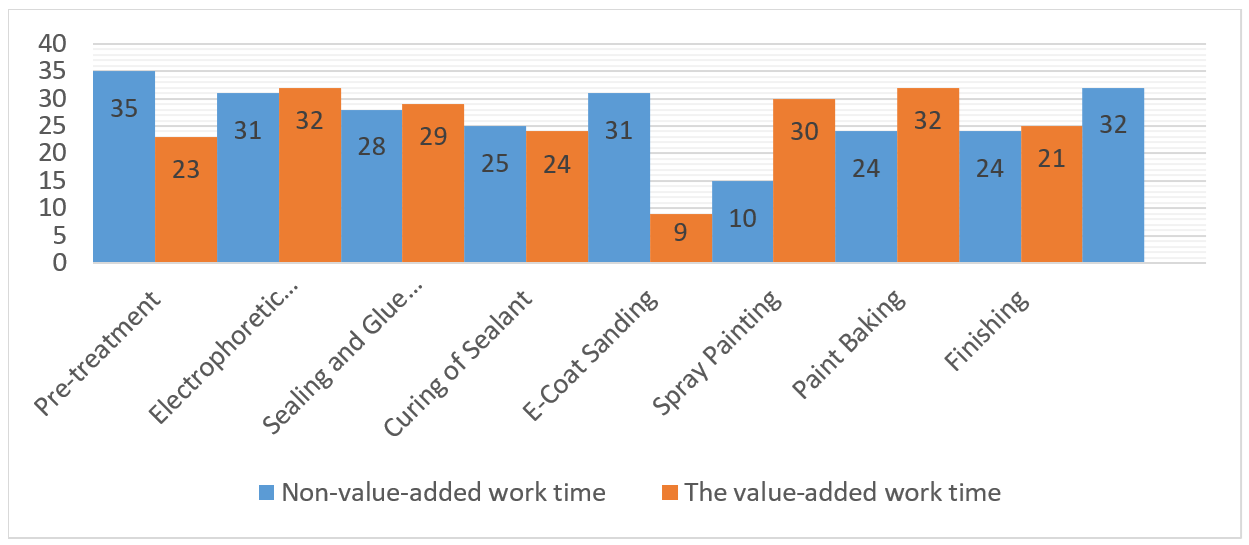

Supplement: S5 Fig — (TIF) [file pone.0318253.s005.tif]
