# Supplementary material for: Study on lean production management of new energy vehicle body painting based on the dual perspectives of digital transformation and VSM
Source: PLoS One. 2025 Feb 14;20(2):e0318253. doi: 10.1371/journal.pone.0318253 (PMC11828361; doi:10.1371/journal.pone.0318253)
Supplement: S1 Table — (DOCX) [file pone.0318253.s006.docx]

| work Content | Operation Time (min) | Non Operation Time (min) | Number of Workers |
| --- | --- | --- | --- |
| Pre-treatment | 23 | 35 | 3 |
| Electrophoretic Drying | 32 | 31 | 1 |
| Sealing and Glue Injection | 34 | 28 | 2 |
| Curing of Sealant | 24 | 25 | 1 |
| Electrophoretic Coating Sanding | 9 | 31 | 1 |
| Spray Painting | 42 | 44 | 4 |
| Paint Baking | 32 | 24 | 1 |
| Finishing | 25 | 24 | 3 |
| Storing | - | 32 | - |
| Total | 221 | 274 | 16 |
