# Supplementary material for: Study on lean production management of new energy vehicle body painting based on the dual perspectives of digital transformation and VSM
Source: PLoS One. 2025 Feb 14;20(2):e0318253. doi: 10.1371/journal.pone.0318253 (PMC11828361; doi:10.1371/journal.pone.0318253)
Supplement: S2 Table — (DOCX) [file pone.0318253.s007.docx]

| No. | Category | Issues Identified | Issue Analysis | Optimization Principles |
| --- | --- | --- | --- | --- |
| 1 | Waiting | Idle Time Due to Work Stoppage,  Waiting for Materials,  Inefficient Equipment Configuration, Surplus Labor | The sealing and glue injection process has a long cycle time, there is an uneven workload between workstations, and there is a time imbalance between the preceding and following processes. Different production processes have relatively long waiting times, leading to gaps between process times and resulting in excessive waste in the production process. | Eliminate excess time, reorganize workloads, and streamline staff. |
| 2 | Transportation | Wasteful Use of Space,  Irrational Production Layout, Problematic Production Sequence Combination | The workshop layout did not fully consider the sequential and continuous nature of the processes, resulting in an irrational production layout. The painting workshop has many production processes, and the layout of the production platform did not adopt a layout method conducive to flow production, which increased the cost of material transportation. | Eliminate excess space, consolidate production processes, and reorder production. |
| 3 | Over-Processing | Low Production Efficiency, Operating According to Past Habits Without Improvement | The resource and working time allocations for each process in the painting production flow are unbalanced, with an excess of personnel and insufficient training, leading to low production efficiency. The production layout is not rational, and the long waiting times result in processing waste. | Eliminate excess time, rearrange production layout, and simplify operators. |
| 4 | Unused Operation | Operations That Do Not Generate Added Value, Operations with Low Productivity | The production line processes have long waiting times, resulting in waste from useless operations in the production process. The painting process has a long cycle time, with lengthy process times and unstable quality. | Simplify useless operations and reschedule beats. |
| 5 | Defects | Waste of Raw Materials, Waste from Rework or Reproduction | Issues with suppliers, inadequate training, and incomplete quality standards lead to waste from defects. | Eliminate material waste. Rearrange quality standards. |
| 6 | Overproduction | Overproduction, Interference with the Uneven Production Flow | Surplus personnel, surplus equipment, and excessive production planning lead to waste from overproduction. | Consolidate production processes, rearrange production schedules, and simplify operators. |
| 7 | Inventory | Inventory Backlog, Excess Production Leading to Unsold Stock | An inadequate system for leveling production, irrational equipment configuration, and production ahead of schedule can all lead to waste from excess inventory. | Eliminate redundant equipment and reschedule production. |
