# Supplementary material for: Study on lean production management of new energy vehicle body painting based on the dual perspectives of digital transformation and VSM
Source: PLoS One. 2025 Feb 14;20(2):e0318253. doi: 10.1371/journal.pone.0318253 (PMC11828361; doi:10.1371/journal.pone.0318253)
Supplement: S3 Table — (DOCX) [file pone.0318253.s008.docx]

| Station Name | Operation Time Before Optimization (min) |
| --- | --- |
| Door Fixture Change | 16 |
| Coarse Sealing Glue | 5 |
| Coarse Sealing Repair Glue | 5 |
| Fine Sealing Glue | 4 |
| Fine Sealing Repair Glue | 4 |
| Total Time | 34 |
