# Supplementary material for: Study on lean production management of new energy vehicle body painting based on the dual perspectives of digital transformation and VSM
Source: PLoS One. 2025 Feb 14;20(2):e0318253. doi: 10.1371/journal.pone.0318253 (PMC11828361; doi:10.1371/journal.pone.0318253)
Supplement: S7 Table — (DOCX) [file pone.0318253.s012.docx]

| No. | Improvement Objective | Original Production Data | Current Production Data | Analysis Calculation Result |
| --- | --- | --- | --- | --- |
| 1 | Value-added operation time | 221 min | 200 min | Increase 9.5% |
| 2 | Non-value-added operation time | 274 min | 240 min | Reduction 12.4% |
| 3 | Production process value-added ratio | 44.6% | 45.5% | Increase 0.9% |
| 4 | Production efficiency | 675 units/month | 850 units/month | Increase 26% |
| 5 | Production personnel | 16 persons | 13 persons | Reduction 18.8% |
| 6 | Production quality | yield rate 89% | yield rate 97% | Increase 8% |
| 7 | Exhaust gas purification rate | 32% | 98% | Increase 66% |
